# Clinical efficacy of macrolide antibiotics in *mycoplasma pneumoniae* pneumonia carrying a macrolide-resistant mutation in the 23 S rRNA gene in pediatric patients

**DOI:** 10.1186/s12879-024-09612-6

**Published:** 2024-07-31

**Authors:** Mengyuan He, Junfeng Xie, Pu Rui, Xiaoyu Li, Min Lai, Hongman Xue, Chun Chen

**Affiliations:** Pediatric Hematology Laboratory, Division of Hematology/Oncology, Department of Pediatrics, The Seventh Affiliated Hospital of Sun Yat-Sen University, Shenzhen, China

**Keywords:** Mycoplasma pneumoniae pneumonia, Pediatrics, Macrolide-resistant, Targeted next-generation sequencing

## Abstract

**Background:**

The global prospective surveillance data showed the re-emergence of mycoplasma pneumoniae pneumonia (MPP) in Europe and Asia after the coronavirus disease 2019 pandemic. We sought to observe the effect of macrolide antibiotics in the treatment of MPP carrying a macrolide-resistant mutation gene and the potential of targeted next-generation sequencing (tNGS) as a front-line diagnostic in MPP patients.

**Methods:**

The baseline characteristics of 91 children with MPP hospitalized from January to October 2023 were retrospectively analyzed. They were divided into two groups according to whether carrying the macrolide-resistant mutation or not. The logistic and linear regression analyses were used to determine whether the mutation was a standalone predictive predictor of the duration of fever and hospital length of stay.

**Results:**

First, no patients had a fever for ≥ 7 days after macrolide treatment. But length of stay and hormone concentration were significantly different between the two groups (*P* < 0.05). There were also no statistical association between the mutation and the duration of fever and hospital length of stay.

**Conclusion:**

Macrolides can be administered to MPP children carrying a macrolide-resistant mutation. tNGS can be seen as a front-line diagnostic in MPP.

## Background

*Mycoplasma pneumoniae* pneumonia (MPP) is currently a significant pathogen in community-acquired pneumonia (CAP) in children, particularly those ≥ 5 years of age [1]. It has a distinct seasonality that peaks in the winter after rising gradually from the summer through the fall [2]. The global prospective surveillance data show the re-emergence of MPP in Europe and Asia more than 3 years after the coronavirus disease 2019 pandemic [3]. Although MPP is mostly benign or asymptomatic, it can occasionally progress into a serious condition that poses a considerable risk to life and causes significant organ damage [1]. MPP is detected most frequently and is the major disease-related burden in CAP-hospitalized children [4].

Targeted next-generation sequencing(tNGS), which is based on ultra-multiplex polymerase chain reaction (PCR) amplification and high-throughput sequencing technology, is more rapid and economical than metagenomic next-generation sequencing [5, 6]. It can achieve an early diagnosis of respiratory infection, including MP [5].

Macrolides are currently recommended as first-line treatment of MPP [7]. However, cases of macrolide resistant MPP (MRMPP) have increased gradually in recent years and now exceed 90% of all MPP cases [8]. Tetracyclines and fluoroquinolones are effective for the treatment of MRMP [7, 9]. However, the use of these medications in pediatric patients has raised concerns due to tetracyclines’ potential to produce enamel hypoplasia and tooth discoloration in children under 8 years of age, as well as the irreversible arthropathy in children induced by fluoroquinolones [9]. Therefore, children with MPP largely continue to receive macrolides.

In addition, three different types of point mutations in domain V of 23s rRNA have been detected in MRMPP, i.e., A2063G, A2064G, A2063C, A2063T, and A2064C, with A2063G accounted for 99.0% of all cases [8, 10]. Currently, the macrolide resistance is determined by measuring mutations at the above loci in clinical practice, but the resistance status detected is not fully consistent with the clinical efficacy. This paper sought to evaluate the clinical efficacy of macrolide antibiotics in MPP pediatric patients carrying a mutation in the 23 S rRNA gene detected by tNGS.

## Materials and methods

### Study population

Pediatric patients who were hospitalized for MPP at a tertiary referral university hospital in Shenzhen, China, from January to October 2023 were enrolled in this retrospective cohort study. Since this study was non-interventional and retrospective in nature, the ethical committee waived the need to collect written informed consent from patients or their legal guardians.

Patient eligibility for enrolment was as follows: (1) under 18 years of age; (2) Diagnosed as MPP according to *Diagnosis and Treatment Guidelines for Mycoplasma Pneumonia in Children* (2023) [7], (3) receiving a macrolide treatment initially after admission, (4) undergoing tNGS of the bronchoalveolar lavage fluid (BALF) and other pertinent samples available for standard procedures ≤ 48 h after admission.

Meanwhile, exclusion criteria included the following: (1) underlying lung tumors, bronchiectasis or tuberculosis; (2) diseases such as severe malnutrition, chronic cardiac, congenital disease, or hematopoietic stem cell transplantation performed at ≤ 90 days; (3) received glucocorticoid, tetracyclines, or quinolones before admission; (4) > 18 years of age; (5) received tetracyclines after admission; and (6) departure without consulting a doctor.

###  Mycoplasmae pneumonia and macrolide-resistant gene detection [5, 6]

Anti-MP IgM titrations were performed at the time of admission. At the same time, tNGS was completed once BALF and other pertinent samples were obtained. Nucleic acid was automatically extracted using the King Fisher flex (Guangzhou Jinqirui Biotechnology Companies) and then amplified by PCR using a multiplex primer set and internal process control primers. Target capture and library creation were accomplished via purification using magnetic beads, and the qualified purified pooling libraries were then sequenced on the KM MiniSeqDx-CN high-throughput sequencing platform. Automatic analysis was performed using the tNGS Data Analysis Management System Version 2.5.0. (Guangzhou Jinqirui Biotechnology Companies).

Amplification of the domain V of the partial 23 S rRNA gene by nested PCR and DNA sequencing was carried out subsequently to find point mutation loci (A2063 G/C, A2064 G/C, and C2617 G/A) that induced macrolide resistance if the real-time PCR analysis yielded a positive result.

Depending on whether the patients had a mutation in the 23 S rRNA gene, they were split into two groups: MS patients were classified as having no mutation in the 23 S rRNA gene, while MR patients had the mutation.

###  Data collection

The following information was collected for all enrolled patients from the electronic medical records: (1) demographic parameters, including age, sex, and body mass index; (2) clinical symptoms and physical signs, such as fever, cough, and labored breathing; (3) laboratory parameters and radiographic features, including MP IgM antibody concentration and white blood cell count as well as the levels of C-reactive protein, lactate procalcitonin, dehydrogenase, alanine aminotransferase, aspartate aminotransferase, and pulmonary consolidation; (4) tNGS results, such as sequence number of MP, and resistance point mutations in domain V of 23 S rRNA; and (5) medical history and hospital stay, including days of macrolides therapy.

###  Definition

Macrolide-unresponsive MPP was defined as persistent fever and worsening in the degree of pneumonic lesions on chest radiographs after 3 days of standard treatment with macrolides [10].

Discharge criteria were defined as follows [11]: clinical stability, normal body temperature for more than 24 h, and they could be switched to oral medication, without complications that required further management, and no mental disorders.

###  Statistical analysis

To evaluate whether the continuous variables had a normal distribution, the Shapiro-Wilk test was performed, and results were given as mean ± standard deviation values and compared with an independent-samples t test if they were followed or as median (interquartile range) values and compared with a Wilcoxon rank-sum test if they were not followed normal distribution. The chi-square test was used to compare categorical variables reported as numbers and percentages. Logistic and linear regression analysis was used to determine whether the point mutations in domain V of 23 S rRNA were a standalone predictive predictor of the duration of fever and length of stay. Baseline variables that were clinically relevant or had a *P* < 0.2 in the univariate analysis were included in the multifactorial regression model. The inclusion variables were carefully selected to ensure the simplicity of the final model, given the number of available events. The software IBM SPSS Statistics (version 25.0; IBM Corporation, Armonk, NY, USA) was used to carry out all analyses. *P* < 0.05 was deemed statistically significant.

##  Results

**Fig. 1:**
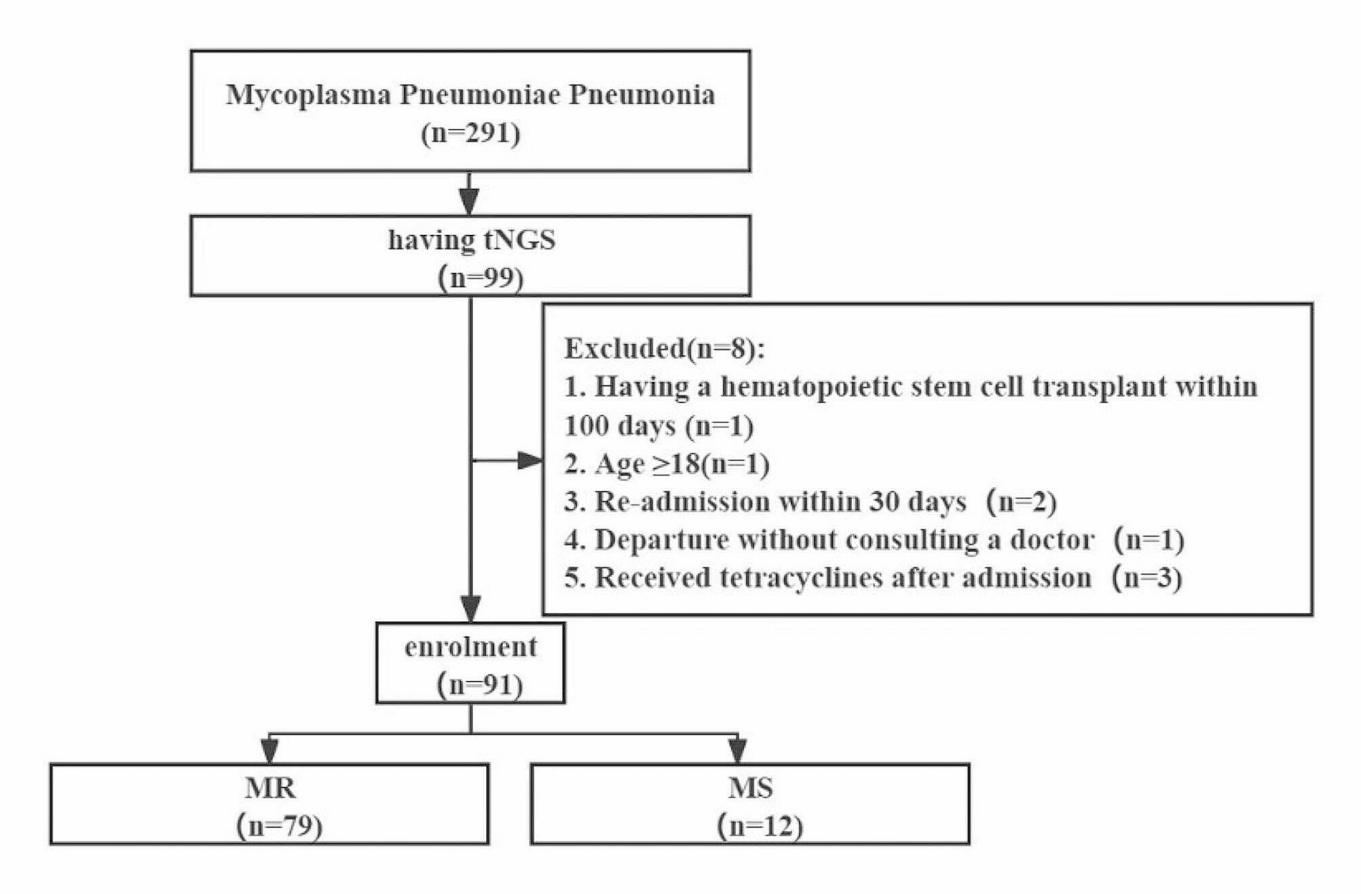
Flowchart of the study population. tNGS, target next-generation sequencing. MR, patients appeared a mutation in the 23 S rRNA gene. MS, patients without mutation in the 23 S rRNA gene

Based on the inclusion and exclusion criteria, 91 individuals were finally recruited into this study, including 79 (86.81%) with a 23 S rRNA mutation and 13 (14.29%) without this mutation, as seen in Fig. 1. Azithromycin was the only macrolide antibiotic used in this study. No patients had a fever for ≥ 7 days after macrolide treatment.

###  Baseline characteristics of the study population

With a median age of 6.29 (4.63–7.58) years, 66.09% of MR patients (50.63% male) were ≥ 5 years of age; with a median age of 5.92 (2.66–7.07) years, 58.33% of MS patients (50.00% male) were ≥ 5 years of age. There was no difference in body mass index, clinical symptoms, or physical signs between the two groups (*P* > 0.05). The mutations in the 23 S rRNA gene of MR patients were all located at A2063G (79/79,100%). However, the length of stay and hormone concentration were significantly different between the two groups (*P* < 0.05) (Table 1). There were no significant differences in laboratory characteristics and radiological features between the two groups (*P* > 0.05 for all), as reported in Tables 2 and 3.

**Table 1:** Clinical characteristics

	MR(*n* = 79)	MS(*n* = 12)	*P*
Age, years	6.29(4.63,7.58)	5.92(2.66,7.07)	0.519
<5y	26(32.91%)	5(41.67%)	0.788
≥5y	53(67.09%)	7(58.33%)	
Male	40(50.63%)	6(50.00%)	0.967
BMI, kg/m^2^	15.20(14.11,16.14)	15.65(13.60,18.08)	0.538
Fever	77(97.47%)	10(83.33%)	0.083
Cough	77(97.47%)	12(100.00%)	1.000
Labored breathing	8(10.13%)	1(8.33%)	1.000
Retraction sign	7(8.86%)	0(0.00%)	0.588
Moist rales	53(67.09%)	10(83.33%)	0.423
Wheezing rales	9(11.39%)	1(8.33%)	1.000
SMPP	48(60.76%)	5(41.67%)	0.211
Pre-macrolide fever duration, days	5.00(4.00,7.00)	6.00(4.25,7.00)	0.896
> 72 h after macrolide, fever	6(7.59%)	1(8.33%)	1.000
Other antibiotic#	66(83.54%)	9(75.00%)	0.751
Hormone	47(59.49%)	1(8.33%)	0.001
Hormone dose, mg/kg/day	1(0,2)	0(0,0)	0.001
Mutation in the 23 S rRNA gene			
A2063G	79(100.00%)	-	
A2064G	0	-	
A2063C	0	-	
Length of stay, days	8.00(7.00, 10.00)	6.00(5.00,7.75)	0.006

#Antibiotics except tetracyclines, quinolones, and macrolides. BMI, Body mass index. SMPP, sever mycoplasma pneumoniae pneumonia.

**Table 2:** Laboratory parameters

	MR(*n* = 79)	MS(*n* = 12)	*P*
White blood cell, ×10^9^/L	7.29(5.69,8.33)	7.82(6.47,11.11)	0.117
Lymphocytes, %	29.30(22.50,37.60)	36.00(23.45,59.13)	0.283
Neutrophil, %	61.90(49.90,70.60)	56.75(30.25,67.65)	0.248
PLT, ×10^9^/L	257.00(205.00,337.00)	256.00(216.25,426.50)	0.669
CRP			1.000
< 10 mg/L	31(39.24%)	5(41.67%)	
≥ 10 mg/L	48(60.76%)	7(58.33%)	
PCT, ng/ml			0.245
<0.05ng/ml	32(40.51%)	7(58.33%)	
≥0.05ng/ml	47(59.49%)	5(41.67%)	
AST, U/L	36.16(30.32,41.09)	34.33(28.23,38.97)	0.542
ALT, U/L	17.67(14.95,20.57)	17.97(15.95,21.45)	0.333
ALB, g/L	41.12(38.86,43.78)	43.32(40.29,45.64)	0.133
LDH, U/L	284.85(251.88,317.11)	276.35(249.16,319.06)	0.743
CK-MB, U/L	17.63(12.62,25.86)	20.31(16.08,32.45)	0.324
Creatinine, µmol/L	31.92(26.99,37.04)	32.18(26.34,42.56)	0.669
Urea, mmol/L	2.90(2.26,3.71)	2.97(2.37,3.64)	0.643

PLT, platelets. CRP, C-reactive protein. PCT, procalcitonin. AST, alanine aminotransferase. ALT, aspartate aminotransferase. ALB, albumin. LDH, lactic dehydrogenase. CK-MB, Creatine Kinase MB isoenzyme

**Table 3:** Radiological features

	MR(*n* = 73)	MS(*n* = 10)	*P*
Pulmonary consolidation	48(65.75%)	8(80.00%)	0.588
Pleural effusion	1(1.37%)	0(0.00%)	1.000
Lobar atelectasis	0(0.00%)	0(0.00%)	1.000
Pleural thickening	2(2.74%)	0(0.00%)	1.000
Bilateral lung involvement	56(76.71%)	6(60.00%)	0.452

###  23 S rRNA gene mutation and fever duration after macrolide therapy

Dualistic regression analysis was carried out to assess whether 23 S rRNA gene mutation was associated with fever for > 72 h. There was no statistical association between them after adjusting for white blood cell count, bilateral lung involvement, and hormone concentration (odds ratio = 0.19, 95% confidence interval = 0.01–4.51, *P* = 0.306), as seen in Table 4.

**Table 4:** The mutation in 23 S rRNA gene and fever > 72 h after macrolide

	OR	*P*	95% CI	
			Lower	Upper
Mutation in 23 S rRNA gene	0.19	0.306	0.01	4.51
White blood cell, ×10^9^/L	0.64	0.151	0.35	1.18
Bilateral lung involvement	0.43	0.442	0.05	3.69
Hormone	3.17	0.395	0.22	45.17

###  23 S rRNA gene mutation and length of stay

A mutation in the 23 S rRNA gene was not associated with the length of stay as the multiple linear regression analysis (*β* = 4.37, 95% confidence interval = [− 4.82, 13.57], *P* = 0.346).

## Discussion

MP is one of the most common pathogens identified in CAP patients at home and abroad [1, 12], and is more prevalent in children ≥ 5 years of age than those < 5 years of age [2]. In this study, the majority of children in the MR and MS groups (67.9% and 58.33%) were older than 5 years of age. Furthermore, the overall percentage of MRMP has progressively grown in China [8, 13], and MRMP has also emerged in other countries among children [14, 15]. MRMP is strongly associated with the emergence of point mutations in the 23 S rRNA gene [16, 17]. In this study, the A2063G mutation in domain V of 23 S rRNA was identified in all patients. This is a consistent with many population-based epidemiological studies, which revealed that A2063G mutations accounted for ≥ 99.0% of cases [8, 13, 18].

MRMP patients have a longer duration of fever and more prolonged hospitalization than macrolide-sensitive MP patients [18, 19]. However, the mutation in the 23 S rRNA gene showed no statistical association with fever duration following macrolide therapy or the length of hospitalization in this study. Yoon IA.et al also demonstrated that macrolide resistance had no effect on MPP fever duration [20]. We hypothesize this is the result of the following reasons.

First, Deng et al. demonstrated that there was no correlation between radiographic findings and the A2063G mutation in the 23 S rRNA gene of MPP patients [21]. Radiologic findings revealed a determinant effect on the clinical course, such as fever duration of MPP [20]. Hence, the 23 S rRNA mutations may not have an impact on the clinical course of MPP, and MPP patients with these mutations may be not necessarily resistant to macrolides. All of the children with the A2063G mutation in our study received azithromycin therapy and discharged after improvement. Macrolide antibiotics may therefore be used as a therapeutic for MPP patients with the A2063G mutation.

What’s more, macrolides bind to 50 S ribosomes in prokaryotic and eukaryotic organisms to exert their antibacterial activity by preventing nascent peptides from translocating or transpeptidation. However, some articles have suggested that the A2063G mutants had high levels of resistance to erythromycin and azithromycin [22]. MPP is self-limiting disease, and there is growing evidence that macrolide antibiotics are not just antimicrobials but also have immunomodulatory effects [23, 24]. Azithromycin prevents the transcription factors nuclear factor-kappa B or activator protein-1, which may be the reason behind their inhibition of the synthesis of various pro-inflammatory cytokines, including interleukin (IL)-1, IL-6, IL-8, and tumor necrosis factor-alpha [25]. The amelioration of clinical symptoms, including fever, may have been facilitated by the immunomodulatory actions of macrolides, which requires more reports to evaluate the anti-inflammatory effects of macrolides in MRMP.

Last but not least, we cannot overlook the function of hormones. Though it’s still unclear what exactly causes the cytokine and hyperinflammatory reactions that follow lung damage from MP infection [7, 26], some studies have demonstrated that hormones therapy has improved outcomes and prevented disease progression in MPP patients [27, 28]. Hormones were used in about 52.75% patients in this study and the role of hormones cannot be ignored. However, Han et al. suggested that there was no significant difference in clinical or laboratory markers between macrolide-resistant and macrolide-sensitive MPP treated with hormones [29]. Likewise, hormones were not associated with the length of hospitalization or fever in this paper. The reason may be the dosing of hormones [30]. Our population used a low dose of hormones. Further studies on the role of hormones and different doses of hormones in MPP are needed.

There are certain drawbacks to our study. First, the sample-collection period—which spanned just 10 months, from January to October 2023—was brief. However, this is the optimal annual time period for MPP cases, and there was a sizable sample obtained. Second, this was a single-center study and may be deemed inadequate for MPP in general. Multi-center investigations with larger sample are needed. Furthermore, no antimicrobial susceptibility testing was completed. But macrolide-resistant gene were identified by tNGS based on ultra-multiplex PCR amplification and high-throughput sequencing technology. Lastly, we did not compare the efficacy of other antibiotics including tetracyclines and fluoroquinolones, with macrolides in MPP. We’ll carry out relevant research in this area.

## Conclusion

Macrolides can be administered to MPP children carrying a macrolide-resistant mutation in the 23 S rRNA gene. TNGS can be seen as a front-line diagnostic in MPP patients. Future multi-center investigations with larger samples are needed to prove it.

## Data Availability

The datasets used and/or analyzed during the current study are available from the corresponding author on reasonable request.
